# Walking hyponatremia syndrome of inappropriate antidiuretic hormone secretion secondary to carbamazepine use: a case report

**DOI:** 10.1186/s13256-018-1744-6

**Published:** 2018-07-11

**Authors:** Pedro José Palacios Argueta, Guillermo Francisco Sánchez Rosenberg, Alvaro Pineda

**Affiliations:** Facultad de Medicina, Universidad Francisco Marroquín, 6 Avenida 7-55, zona 10, 01010 Guatemala City, Guatemala

**Keywords:** Asymptomatic, Carbamazepine, Hyponatremia, SIADH, Side effect

## Abstract

**Background:**

Severe hyponatremia is rare when carbamazepine is used as monotherapy. It is common to encounter this imbalance in the hospital setting, but rare in the ambulatory one. Here, we present a case of hyponatremia secondary to carbamazepine use in an otherwise asymptomatic patient.

**Case presentation:**

A 44-year-old Guatemalan woman presented to our outpatient clinic with a chief complaint of left knee pain. One month prior, our patient had previously consulted with an outside physician, who prescribed her with 300 mg of carbamazepine, 5 mg of prednisone every 24 hours, and ibuprofen every 8 hours as needed. The symptoms did not resolve and our patient had increased the dose to 600 mg of carbamazepine and 20 mg of prednisone 7 days prior. Our patient complained of left knee pain, fatigue, and bilateral lower limb cramps. No pertinent medical history was recorded and her vital signs were within normal limits. A physical examination was non-contributory, only multiple port-wine stains in the upper and lower extremities associated with mild hypertrophy of the calves, more prominent on the right side. Laboratory studies revealed: a serum sodium level of 119 mmol/L, potassium level of 2.9 mmol/L, thyroid-secreting hormone of 1.76 mIU/m, thyroxine of 14.5 ng/dL, and serum osmolality at 247 mmol/kg. No neurologic or physical disabilities were recorded. In the emergency department, her electrolyte imbalance was corrected and other diagnostic studies revealed: a urinary sodium level of 164 mmol/L and osmolality at 328 mmol/kg. Our patient was diagnosed with syndrome of inappropriate antidiuretic hormone secretion secondary to carbamazepine use, hypokalemia secondary to corticosteroid therapy, and Klippel-Trénaunay-Weber syndrome. Carbamazepine was discontinued, fluid restriction ordered, and hypokalemia was corrected. One week after discharge, our patient no longer felt fatigued, the cramps were not present, and her left knee pain had mildly improved with acetaminophen use and local nonsteroidal anti-inflammatory cream. Electrolyte studies revealed a sodium level of 138 mmol/L, potassium level of 4.6 mmol/L, and serum osmolality at 276 mmol/L.

**Conclusions:**

Hyponatremia can be misdiagnosed if not recognized promptly; suspicion should be high when risk factors are present and the patient has been prescribed antiepileptic drugs. Presence of mild symptoms such as fatigue or dizziness should lead to suspicion and subsequent laboratory testing. Patients can suffer from neurologic complications if the imbalance is not corrected.

## Background

Hyponatremia is defined as a serum sodium (Na^+^) level of < 136 mmol/L, and is considered significant when levels are between 115 and 125 mmol/L [1]. Acute hyponatremia (less than 48 hours) can cause neurologic complications such as seizures and coma and it necessitates urgency of care to prevent complications [1, 2]. Most of the patients develop symptoms when serum Na^+^ concentration is 119 ± 9.1 mmol/L [3]. Asymptomatic hyponatremia is common in patients with carbamazepine use; some of the few symptoms are dizziness and somnolence, which are easily neglected in the clinic [4]. The main mechanism by which carbamazepine causes hyponatremia is by increased antidiuretic hormone (ADH) secretion, but it has also been related to increased sensitivity of the renal tubules to ADH activity as well as increased aquaporin 2 channel expression in the renal tubule [5]. In the following case, we present the case of a patient with asymptomatic hyponatremia secondary to recent carbamazepine use. Our patient had self-medicated to twice the original dose of carbamazepine and presented with Na^+^ levels of 119 mmol/L and a chief complaint of left knee pain.

## Case presentation

**Fig. 1:**
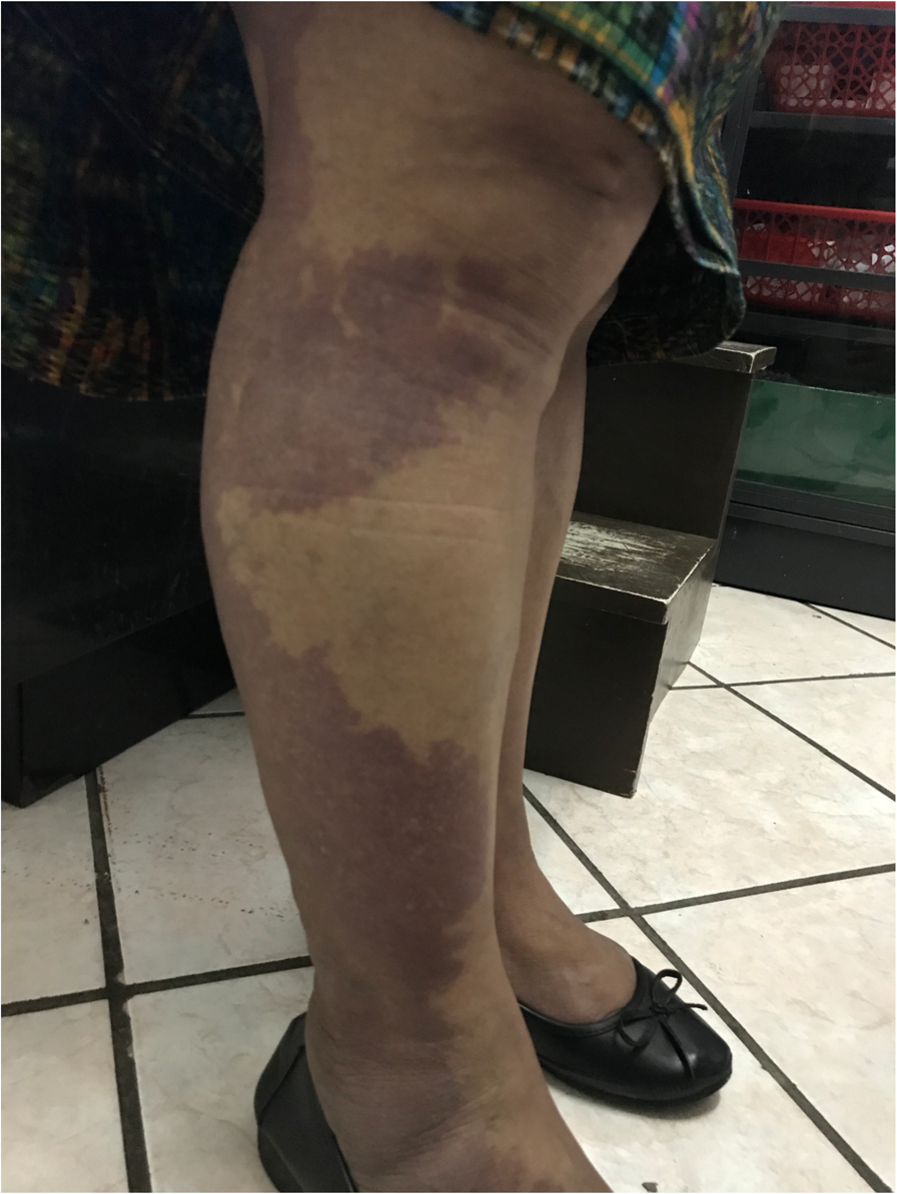
Bilateral lower limb port wine stains

A 44-year-old Guatemalan woman presented to our outpatient clinic with a chief complaint of left knee pain for the last 6 months. One month prior our patient had consulted with an outside physician, who prescribed her with 300 mg of carbamazepine, 5 mg of prednisone every 24 hours, and ibuprofen every 8 hours as needed. The symptoms did not resolve and our patient increased the dose to 600 mg of carbamazepine and 20 mg of prednisone 7 days before consulting. She suddenly interrupted prednisone 3 days before consulting, because she felt this was making her pain worse. At the consultation, our patient complained of left knee pain, fatigue, and bilateral lower limb cramps. No pertinent medical, surgical, allergic, family, and psychosocial history was recorded and her vital signs were within normal limits (blood pressure of 118/64 mmHg; heart rate of 75/minute; respiratory rate of 14 beats/minute; temperature at 98.9 °Fahrenheit). Our patient was on day 12 of her menstrual cycle. A physical examination revealed mild facial edema, multiple port-wine stains on her upper and lower extremities associated with mild hypertrophy of the calves (Fig. 1), more prominent on the right side with mild edema, the rest of the physical examination was non-contributory. Imaging and routine blood studies were requested. Our patient was seen the next day at the office with the laboratory results (Table 1).

**Table 1:** Laboratory values

Test	Result	Normal values
Na^+^, mmol/L	119	135–145
K^+^, mmol/L	2.9	3.5–5.5
TSH, mIU/mlL	1.76	0.5–4.70
T4, ng/dL	14.5	10–23
Fasting glucose, mmol/L	5.7	< 6.1–6.
BUN, mmol/L	4.4	2.5–8.0
Cr, μmol/L	88.0	</= 106

*Na*^*+*^ sodium, *K+* potassium, *TSH* thyroid-stimulating hormone, *BUN* blood urea nitrogen, *Cr* creatinine

Our patient presented with only a complaint of fatigue at this visit, however, no neurologic or physical disabilities were recorded, and her vital signs were within normal limits.

Our patient was sent to the emergency department (ED) for electrolyte imbalance correction and for other diagnostic studies. In the ED, a 12-lead electrocardiogram did not show any electric abnormalities, her urinary Na^+^ (UNa^+^) was 164 mmol/L (normal 40–220 mmol/L), and osmolality 328 mmol/kg (50–1200 mmol/kg). No carbamazepine serum levels were recorded due to lack of reactive in the hospital. Our patient was diagnosed with syndrome of inappropriate antidiuretic hormone secretion (SIADH) secondary to carbamazepine use, hypokalemia secondary to corticosteroid therapy, and Klippel-Trénaunay-Weber (KTW) syndrome. Patient orders were to discontinue carbamazepine, fluid restriction to 1.5 L/day and intravenous potassium (K^+^) slow correction. She was discharged 3 days later with Na^+^ 128 mmol/L, K^+^ 4.6 mmol/L, and serum osmolality 265 mmol/kg. Our patient was seen in the office 1 week after discharge: she no longer felt fatigued, her cramps were not present and her left knee pain had mildly improved with acetaminophen use and local nonsteroidal anti-inflammatory drug (NSAID) cream. Her left knee pain was attributed to the KTW syndrome. She was not taking carbamazepine and prednisone at the time of the follow-up. Electrolyte studies revealed Na^+^ 138 mmol/L, K 4.6 mmol/L, and serum osmolality at 276 mmol/kg.

## Discussion

Severe hyponatremia is rare when carbamazepine is used as monotherapy [6], however if it does develop, it is most common in the first 3 months [7], with a median onset of 38.5 days [8]. Our patient had been on carbamazepine therapy for less than 30 days. The reason for her being prescribed carbamazepine is unknown, but most likely secondary to chronic pain. Our patient increased the dose herself to twice the originally prescribed amount; this might have prompted the exacerbation of the sodium imbalance, even though baseline sodium levels were not recorded. Several risk factors have been associated with the development of carbamazepine-induced SIADH, such as female sex, being over 40 years of age, use of other medications known to cause hyponatremia, menstruation, psychiatric condition, surgery, psychogenic polydipsia, low baseline serum Na^+^ levels, and hypothyrodism [3, 4, 7, 8]. In this case, our patient’s only risk factors were age above 40 years and female sex. It is unknown if KTW syndrome is a risk factor for SIADH or hyponatremia due to the rarity of this pathology. The only major symptom our patient showed was fatigue. Other factors have been attributed to the lack of symptoms in cases of slowly developing hyponatremia, as decreased uptake of taurine aims to adapt to the decrease in extracellular osmolality [9]. In the work by Pliquett *et al.* [9], some of the symptoms in patients with moderate hyponatremia include headache and modest nausea. Our patient failed to show these symptoms, which can be attributed to her young age and the few risk factors she presented with, but if many more cases are reported, fatigue may be added to this list and can be one sign to look for hyponatremia in patients prescribed with carbamazepine. Treatment of this pathology includes fluid restriction and management of the underlying cause [10]. The use of demeclocycline and lithium is not recommended due to the increased risk of harm versus the benefits of treatment [11]. Our patient showed good response to management with fluid restriction and discontinuation of carbamazepine therapy. Predictors of poor response to therapy are serum osmolality > 500 mmol per kg, 24 hour urine output > 1.5 L, increase of > 2 mmol of serum Na^+^ in 24 hours, and a serum Na^+^ level less than the sum of UNa^+^ and K^+^ levels [12]. Our patient only presented with the poor response predictor of serum Na^+^ levels of 119 mmol/L, which was still less than the sum of UNa^+^ and K^+^ levels, but fortunately for our patient, she responded well to therapy. We did not request cerebral imaging studies due to the lack of neurologic symptoms and the lack of pertinent history of trauma or a psychiatric condition.

## Conclusions

Hyponatremia can be misdiagnosed if not recognized promptly; suspicion should be high when risk factors are present and the patient has been prescribed antiepileptic drugs. Presence of mild symptoms such as fatigue or dizziness should lead to suspicion and subsequent laboratory testing. Patients can suffer from neurologic complications if the imbalance is not corrected. At the moment, there is no literature available associating KTW syndrome as a risk factor for this condition. Therapy should be limited to fluid restriction and discontinuation of the causative agent since the use of other medications is not recommended.

## Abbreviations

ADH: Antidiuretic hormone
BUN: Blood urea nitrogen
Cr: Creatinine
ED: Emergency department
K^+^: Potassium
KTW: Klippel-Trénaunay-Weber
Na^+^: Sodium
NSAID: Nonsteroidal anti-inflammatory drug
SIADH: Syndrome of inappropriate antidiuretic hormone secretion
TSH: Thyroid-secreting hormone
UNa^+^: Urinary sodium

## Authors’ information

PJ and GF serve as assistant professors of ambulatory medicine in Universidad Francisco Marroquin in Guatemala City and serve as outpatient general physicians at Obras Sociales del Hermano Pedro in Antigua Guatemala. AP is the director of the course and is a retired professor of laboratory medicine from Mayo Clinic, Rochester, MN, USA.
